# Registered report protocol for an e: Health motor skills and physical activity intervention in early childhood education centers- e: Motor skills At Playtime (MAP)

**DOI:** 10.1371/journal.pone.0308047

**Published:** 2024-08-29

**Authors:** Kara K. Palmer, Lu Wang

**Affiliations:** 1 School of Kinesiology, University of Michigan, Ann Arbor, Michigan, United States of America; 2 School of Public Health, University of Michigan, Ann Arbor, Michigan, United States of America; Ahvaz Jundishapur University: Ahvaz Jondishapour University of Medical Sciences, ISLAMIC REPUBLIC OF IRAN

## Abstract

**Background:**

Children have alarmingly low levels of competency in fundamental motor skills (FMS) and high levels of physical inactivity. e:health interventions, interventions delivered electronically, are useful tools for intervention in the home through parents, but less is known about the effects of these interventions in early childhood education centers or settings. Therefore, we created the Motor skills At Playtime (MAP) e:health intervention (e:MAP) to be delivered in an early childhood education setting. The goals of this pilot study on e:MAP are to (1) determine the intervention effects on children’s FMS and physical activity and (2) explore the teachers’ perceptions and ability to facilitate e:MAP.

**Methods:**

This pilot study uses a pretest/posttest randomized cluster control design. We will recruit at least 64 children (3.5–5 years of age) enrolled in a single early childhood education center. Children will be randomly assigned at the level of the classroom to an e:MAP group (n~30) or a control group (n~30). Children in classrooms assigned to e:MAP will complete an 8-week intervention. We will collect measures of child FMS and physical activity, and teacher’s perceptions of the program before (pretest) and after the intervention (posttest). FMS measures include process (Test of Gross Motor Development-3rd Edition) and product-oriented scores. Physical activity will be assessed using a 7-day accelerometer wear protocol. Teachers’ perceptions will be assessed through a brief survey. Lastly, we will collect data on teachers’ ability to facilitate e:MAP through a daily survey.

**Potential significance:**

This study will yield novel insights into the effectiveness and feasibility of a health intervention in an early childhood education setting. Results from this work will expand our knowledge of how to harness e:health modalities, which have the potential to significantly expand the distribution and scalability of FMS interventions.

## 1. Introduction

Physical activity (PA) is an essential component of a healthy lifestyle yet alarmingly, only 20% percent of adolescents meet global physical activity recommendations [1]. The lack of PA is concerning as physical inactivity is linked to the manifestation of non-communicable diseases [2]. There are currently predicted to be 499.2 million new yet preventable noncommunicable diseases occurring by 2030 if physical inactivity is not addressed [2]. Consistent physical inactivity is seen across the lifespan, even in children. Currently, less than half of young children (i.e., children 3–5 years old) are meeting recommended PA guidelines [3, 4], and only 11.26% of children this age are meeting 24-hour movement guidelines, which include daily PA recommendations [5]. More locally, it is reported that in the state of Michigan, only 23% of children currently meet national PA recommendations [6]. Therefore, low levels of PA are both a global and local concern, and it is paramount we address this problem.

Children need competency in fundamental motor skills (FMS) to be active, but these skills are frequently overlooked in intervention and programmatic approaches to improve PA. FMS are gross motor skills that allow children to propel or manipulate their bodies and objects in space (e.g., running, throwing), and are building blocks for more advanced movement and engagement in PA [7–10]. FMS are reciprocally and inseparably linked to current and future PA [11–17]. There is a common misconception that these skills naturally emerge. However, evidence supports that FMS are not learned in the absence of instruction [18], and approximately 77% of children have delayed or impaired motor skills [19]. While a child at any age with a motor delay or impairment is concerning, the early childhood years (3–5 years) are an important developmental period and are part of the critical window of development for FMS [7–9]. Due to the developmental nature of these skills, skills learned during this period have the capacity to support future health, whereas failing to learn these skills at a young age could have negative ramifications for future health.

Despite this, young children are failing to learn FMS [18–21]. which is alarming from a public health and developmental perspective. Evidence-based and theoretically grounded interventions are needed to address these deficits. These interventions should capitalize on the conceptual and logical link between FMS and PA whereby engaging in FMS is engagement in PA and gaining FMS competency supports future PA engagement. Encouragingly, successful interventions have been used to teach FMS to young children [18, 22–26]. These interventions vary in terms of dose and implementation strategies; however, meta-analytic data supports that most successful interventions are shorter than 6-months in duration and implemented by non-school personnel (aka researchers) [22]. While it makes sense that individuals with expertise in motor development would implement highly effective interventions, limiting interventions to those implemented by personnel external to the early childhood education center or school setting limits the distribution of these programs. Therefore, there is a need to transition intervention efforts to those that can be completed within school or other settings (e.g., home) without the researcher’s presence. In the school setting, teachers likely play an important role in intervention implementation and outcomes. A recent review found that teachers can effectively implement physical education interventions/programs with small to moderate improvements in children’s (ages 5–12 years) FMS [27]. We have also seen several groups working to capitalize on the potential for teachers and deliver FMS interventions through teachers. For example, there is recent work to translate the Children’s Health Activity Motor Program from a researcher-implemented program in early childhood centers [28] to a school- or program-staff-led intervention in afterschool settings, a train-the-trainer model [29]. Even though this work is with older children (i.e., elementary school), physical educators or educators with some background in movement, and still includes some ongoing research support, this work is encouraging and demonstrates the timeliness of research efforts to create interventions that fit within the school setting.

Early childhood education centers have unique challenges that must be addressed when creating interventions that could be completed in this setting without external support. In the United States, research supports that teachers in early childhood education have little knowledge about movement guidelines and the importance of structured movement and FMS-building movements in early childhood [30]. This finding mirrors meta-analytic work from a more global perspective [31]. In their systematic review and meta-analyses of >2,300 educators in >260 centers, Jerebine et al [31] reported the two largest barriers early childhood educators face in implementing movement programs are opportunity and capability. Therefore, interventions created for this population should be designed to address these barriers. These programs should fit within current gross motor opportunities within the school and provide educators with training and resources whilst simultaneously limiting the additional burdens on teachers or school staff.

To address these barriers while still creating evidence-based interventions, we propose a change in the modality in which the intervention is delivered. e:health and m:health interventions- interventions that are delivered electronically (e:health) or with the use of mobile devices (m:health) are powerful tools and could be highly effective modalities to deliver motor skill interventions in school settings. These interventions may be effective at improving children’s physical activity behaviors [32, 33], and, currently, there are several m:health interventions that have improved health outcomes in children such as weight outcomes [34] and motor skills and physical activity in young children [35, 36]. The Promoting Lifelong Activity in Youth (PLAY) project is an app-based intervention delivering 12 hours of theoretically grounded intervention content to children through their parents [35, 36]. Families in the PLAY project reported improvements in the children’s motor skills [36]. Additional research in schools also supports that school-based interventions delivered using these e:health or m:health modalities have positive effects on children’s health behaviors including PA and fruit and vegetable consumption [37]. Thus, e:health and m:health modalities are effective, and more research on how these modalities could be used to deliver FMS and PA interventions in early childhood education settings is needed.

Researchers creating interventions should also carefully consider theoretical underpinnings to be used in their design. Three of the main theories used in FMS interventions include Dynamic Systems Theory, Achievement Goal Theory, and Self-Determination Theory [26]. While there is some evidence to support that theoretically, grounded and atheoretical settings interventions are equally effective for improving motor outcomes [26], researchers should consider additional benefits of grounding programs in theory. Integrating Achievement Goal Theory could lead to increased persistence in the face of difficulty [38], positive attitudes toward learning [39, 40], or belief in the direct positive link between effort and outcome [39, 41]. Therefore, it is arguably better to select and use a theory within the design of intervention programs to help elicit not only FMS gains but also improvements in non-motor outcomes that could advantage the learner. One of the most well-researched theoretical approaches in FMS interventions is integrating Achievement Goal Theory by teaching skills in a mastery-motivational climate [23]. Interventions using mastery-motivational climates have immediate and sustained benefits on children’s movement and health outcomes [23]. These programs also improve psychosocial outcomes (e.g., self-regulation and perceived competence) linked to current health and school readiness [42–44].

Interventions using Achievement Goal Theory interventions vary in terms of dose (ranging from 9 weeks to 9 months [23]. To the best of our knowledge, all but one intervention [45] designed with Achievement Goal Theory has been implemented by trained researchers. In this study, a non-motor expert who was still an external person went into the schools and delivered the program, which was delivered during children’s 30-minute outdoor free play period 3 days a week for 15 weeks (1,350 minutes total) [45]. The nonmotor expert completed a 6-hour training prior to the intervention and was responsible for demonstrating all motor skill stations as well as setting up skill stations on the playground. The non-motor expert did not provide any additional feedback or instruction on the playground. Due to the instructional differentiation between the implementation of this program and other work using Achievement Goal Theory, the authors reported this program a a pseudo-mastery climate. After the intervention, children who completed this program exhibited positive changes in their locomotor skills. This work also reported that the intervention was highly feasible and was implemented correctly the majority of the time (>95% of sessions) [45]. Therefore, FMS interventions implemented by non-motor experts in a pseudo-mastery climate might be an important approach to addressing current limitations of the scope of sustainability and scalability. More research in this area is warranted.

There is a particular need for interventions to promote FMS and PA in children who are at risk for current or future health disparities. There are several risk factors associated with poorer FMS and PA in preschoolers, including being from a family with lower SES or an ethnic minority [19, 46–50]. This discrepancy is alarming as these preschoolers are also at risk for other health disparities, including unhealthy weight status [51, 52], inflammation [53], and poorer future cardiovascular health [49]. Additionally, children from low-income families are less prepared for school and underperform compared with children from non-low-income families [54–56]. Movement is central or strongly associated with all these outcomes [57–63]. supporting the need for interventions to promote both FMS and PA specifically in these at-risk populations.

In conclusion, there is a need for FMS interventions that can be implemented in the absence of a researcher’s presence. e:Health and m:health interventions to promote FMS are an emerging and exciting area of research, and more work is needed to examine how these modalities could be used to deliver FMS and PA interventions in early childhood education settings. This research is particularly needed for children who are at risk for future health disparities, such as children in federally subsidized early childhood education centers serving low-income families. This study addresses this need by creating the e:Motor skills At Playtime (MAP) intervention. e:MAP is an FMS intervention grounded in Achievement Goal Theory and is specifically designed to be implemented through an e:health modality within early childhood education settings. The purpose of this pilot study was twofold. First, we will determine the effects of e:MAP on health outcomes, including FMS and PA, in children who are at risk for future health disparities. Secondly, we will gain insight into the usability and potential sustainability of e:MAP within the early childhood education setting by exploring how teachers perceive the program as well as their ability to facilitate the program.

**Goal 1**: Determine the effects of the MAP e:health program on children’s health outcomes (FMS and PA).
**Goal 2**: Explore teachers’ perceptions of and ability to facilitate the MAP e:health program within an early childhood education setting.

## 2. Materials & methods

### 2.1. Sample

All children enrolled in preschool classrooms at a single Head Start/Great Start Preschool in Michigan will be recruited. Based on initial MAP work [45], we completed a sample size calculation using an alpha level of 0.05 and a desired power of 80% when comparing the two groups to detect an effect size of Cohen’s d = 0.8 [64]. This estimated effect size represents a meaningful change in children’s motor skills and is higher than meta-analytic reports of average effects sizes from motor interventions [18, 22]. The calculation yielded a minimum of 54 children needed (27 per group). To account for 20% lost to follow-up, we will recruit 32 children per group. We will try our best to minimize the loss of follow-up, and this extra recruitment will help us maintain statistical power with any unexpected smaller detectable group differences in the outcome. Due to the nature of research in a school setting, children will be randomized at the level of the classroom. We will recruit from a minimum of 6 classrooms.

To be enrolled, children must meet the following at the time of study enrollment: 3.5–5.0 years old, able to understand simple verbal instructions in English, and be physically capable of completing the motor skills assessment. Children who have a physical or intellectual impairment that would inhibit them from successfully completing the motor skill assessment will be excluded.

### 2.1.1 Recruitment

Study recruitment will begin in August 2024 and be completed in three ways: (1) by teachers using approved scripts during school home visits, (2) by the research team during parent/school open house events, and (3) through materials distributed through backpacks during the first month of school. Students will be enrolled on a first-come, first-served basis. If we have recruited 90 children, recruitment will end, and no additional students will be enrolled.

### 2.2. MAP e:Health

#### 2.2.1. Theoretical underpinnings

Motor Skills at Playtime (MAP) is a high-autonomy intervention designed to be implemented within the established gross motor opportunities in schools [45]. MAP is rooted in Achievement Goal Theory [39, 65–67] and is delivered through a pseudo-mastery motivational climate. Specifically, a full mastery climate includes six specific components or structures commonly known as the TARGET structures (task, authority, recognition, grouping, evaluation, and time) [67] (see Table 1). MAP implements four of the six target structures (task, authority, grouping, and time) [45]. The two structures not included (recognition and evaluation) were removed to (1) increase the sustainability and scalability of the program and (2) fit within the m:health modality. This removal is key as we are asking teachers and schools to facilitate, not implement, the program; however, by removing these structures, we recognize we can no longer claim this program as a mastery-motivation climate. Therefore, we have termed this particular implementation as a pseudo-mastery climate. See Table 1 for a full description of the TARGET structures and their application and examples of application in MAP.

**Table 1:** TARGET Structures and their Inclusion in MAP m:health.

Structure	Conceptual Definition	Inclusion in MAP m:health	Example
**T**ask	Design movement tasks and instructional activities to encompass a variety of levels	All activities are designed to incorporate a minimum of three levels of difficulty (e.g., easy, medium, or hard). The videos include a full verbal description of easy and hard activities and invite participants to “choose the easy [insert name of skill], hard [insert name of skill], or something in the middle”.	Audio included on instructional video: “It’s simple to make throwing easier or harder. It is easier to throw at big targets or targets that are close to you. It is harder to throw at small targets or targets that are far away from you. So you can choose. Do easy throws, hard throws, or something in the middle. Choose what is right for you.”
**A**uthority	Shared decision making. Children actively participate in the instructional process	Motor skills are added to the extant gross motor play. Children have authority in how they play during that time. We also encourage teachers to include children when selecting the session number for the day.	Ask the children if they want to do session 1 (throw and run) or session 3 (throw and gallop).
**R**ecognition	Feedback/praise is provided individually and in private	Not included	Not included
**G**rouping	Self-selection of peer interactions	Motor skills are added to the extant gross motor play. Children have the authority to select what peer(s) they play with or if they play by themselves.	Have the children self-select peer groups during extant gross motor play.
**E**valuation	Self- or individual standards for progress	Not included	Not included
**T**ime	Flexibility in schedules; flexible pace of learning	Motor skills are added to the extant gross motor play. Children have the authority to select where they spend their time during the play period.	Children can select to play with MAP stations or continue to play without them. Teachers will report back daily on if the children did or did not play with MAP stations.

#### 2.2.2. Components of e:MAP

The e:MAP program contains two distinct parts: (1) instructional videos and (2) motor skill activities.

Children will view instructional videos in their classrooms prior to engaging in their established gross-motor playtime at the school. This time is typically outdoor recess or free play in a large gym or open space if the weather inhibits outdoor play. The instructional videos contain all instructional elements of the program and are delivered electronically through a private YouTube channel. These videos adhere to tenents of Achievement Goal Theory, such as including a description of how to self-modify the FMS tasks based on the needs of the learners and peer-modeling [67]. In total, there are 48 videos that are classified to the user as sessions. Users are given a suggested order to view the sessions but can self-select their own sessions if they prefer. Each session video is approximately 3 minutes in length and includes a video with two motor skills (one locomotor skill and one object control skill). The skills are first introduced and explained by an adult, and then three skill demonstrations are provided. The first skill demonstration is of the adult correctly executing the skill. The second and third demonstrations are of a preschool-aged boy [5 years] and a girl [3 years]. The order of the child demonstrations is randomly assigned as to which child demonstration is presented first. After the completion of the demonstrations, the user hears an explanation and sees visual prompts on how to adjust the skill practice to make it easier or harder (task of the TARGET structures). The overall user experience within each session was designed to adhere to effective design features within e:health interventions (see Table 2) [68].

**Table 2:** Design Features Associated with Effective e:health Interventions^57^ and their Inclusion in MAP m:health.

Feature	Inclusion and Examples in MAP m:health
Social context and support	All videos include recordings of a person speaking to the audience. The videos also include an adult model interacting with preschool-aged children (one boy [5 years] and one girl [3 years]).
Contacts with intervention	Teachers work with interventionists directly during a 2-hour professional development session before the start of the school year.
Tailoring and targeting	All videos include modeling from a younger [3 year old girls] and older [5 year old boy] model. This allows children at different ages and stages of development to see a model more closely resembling them (tailoring). The videos also include clear verbal instructions for how to practice for each skill could be tailored to suit the needs of the user [see TARGET structure “task” in Table 1].
Self-management	Children are encouraged to monitor their only preferences and current skill levels to engage in the intervention in a meaningful and fun way. [see TARGET structures “Task”, “Grouping” and “Time” in Table 1]
Entertainment	The videos include music and animation as appropriate for a child audience. The in-person sessions that pair with each video are designed for children ages 3–7 years and include age-appropriate and engaging equipment (e.g.., colorful scarves, hula hoops, chalk, etc),
Aesthetics	The videos include color and music as appropriate for young audiences. They also include verbal descriptions and modeling from peers.
Updated information	Teachers will complete a daily facilitation survey. Interventionists will be present at the school four times across the school year (August, October, Feb, and May).
Usability	The videos are all pre-made and ready for use. The corresponding skill stations are predesigned so that all the necessary equipment for each skill is stored in a designated location. Teachers are provided withtwo or three choices of station setups that are possible to use in conjunction with each skill.
Credibility	Schools are provided with an overview pamphlet of the program prior to the start of the school year. This pamphlet includes a list of credentials of the designers of the intervention.
Information architecture	All videos are available through a private YouTube Channel. Participants are allowed to self-navigate through the intervention.
Program exposure	The intervention will be available to the intervention classrooms from October–end of the school year.

The second part of the e:MAP program is the motor activities. Each skill included in the videos has a pre-determined set of two to three possible activities, and the corresponding activities are set up during children’s outdoor recess or free play in a large gym or open space if the weather inhibits outdoor play. Facilitators are provided with all the necessary instructions and equipment to set up all activities. The organization and design of these activities were curated to take less than five minutes to complete all set-up associated with a single session.

#### 2.2.3 Facilitation of MAP

The e:MAP program will be implemented four days a week for 8 weeks (30 min/session, 4 sessions/week, 8 weeks, total 960 minutes). Teachers of classrooms randomly assigned to the MAP condition will facilitate the program. Teachers will be responsible for selecting and playing a session video prior to each gross motor playtime and setting up the corresponding stations. Teachers will also complete a brief survey reporting on the program facilitation (see “2.3.4 Teacher Facilitation”).

#### 2.2.4 e:Health platform

All e:MAP sessions were made available through a private YouTube channel. The full library will be available to teachers the early childhood education centers at the start of the intervention.

### 2.3 Assessments

#### 2.3.1 FMS

We will measure FMS using a battery that yields both process- and product-oriented motor skill scores.

Children will complete the Test of Gross Motor Development, 3rd edition (TGMD-3) [10]. The TGMD-3 is a valid and reliable process-oriented assessment used to measure motor skills in children ages 3 through 10 years. The TGMD-3 measures performance on seven ball skills (1-hand strike, 2-hand strike, dribble, toss, kick, throw, and catch) and six locomotor skills (run, gallop, hop, skip, slide, and jump). Administration and scoring of the TGMD will be done according to the test manual. Children will receive a digital demonstration of the skill before the practice trial [69]. If a child does not understand the specific motor skill during the practice trial, the child will be given a second live demonstration of the skill. All test trials will be coded by an expert coder with >10 years of experience working with the TGMD. Total raw scores for the locomotor subscale (0–44), ball skill subscale (0–56), and total TGMD (0–100) will be calculated.

For five skills- throwing, kicking, standing long jump (SLJ), running, and hopping, we will simultaneously collect product scores. Final scores will include max throwing speed (m/s), max kicking speed (m/s), max jumping distance (cm), max running speed (m/s), and average of max hopping speed on the preferred and non-preferred foot (m/s) [70, 71]. Scores for throwing, kicking, standing long jump (SLJ), and running will be collected from the TGMD-3 test trials [71]. Max hopping speed for the preferred foot will also be assessed from the TGMD-3 test trials, but children will complete two additional hop trials on the non-preferred foot to determine max speed on the non-preferred foot to be used in the final hop score [71].

The final scores used in the main analyses will be the total TGMD raw score, the total summed product score (sum of z-scored product scores) [70]. and a total motor competence score. Motor competence score will be created by standardizing all 13 skills included in the process score (e.g., TGMD-3 skills) and all 5 product scores, adding standard scores, and then scaling from 0 to 100, with 100 representing higher motor competence.

#### 2.3.2 Physical activity

Physical activity will be measured with ActiGraph accelerometers (Actigraph, Pensacola, FL, USA) secured by a hospital band on participants’ non-dominant wrists for one full week (i.e., 5 weekdays and 2 weekend days). The devices will be placed on the child during the school day and set to start recording at midnight. The devices will be removed after seven full days of recording. The devices will be calibrated to collect data at 30 hz, the standard frequency used with accelerometers. Data will be reduced using Actilife software and physical activity behaviors will be determined using cut-points by Johansson et al [72]. The final outcome will be minutes of total and MVPA across the whole day and school day. To be included in the overall physical activity outcome, children will need 3 weekdays + 1 weekend with at least 10 hours of valid wear time[73, 74].

#### 2.3.3 Teacher perceptions

To determine teachers’ perceptions of MAP, we will ask them to complete two surveys- before the intervention (baseline; 7-question survey) and after the intervention (posttest, 9-question survey). Surveys were created for this project but were modeled after previous research studies that examined preschool teachers’ perceptions and experience with classroom-based interventions [75]. Surveys will ask how teachers perceive the following: (1) the importance of the program (n = 2 questions), (2) the ability of the program to help them address their goals regarding child gross motor and physical development (n = 1 question), (3) the appropriateness of the content (n = 2 questions), and (4) the ease of use of the program content (n = 2 questions). The post-test assessment also asks teachers two questions about their beliefs regarding the future use of the program.

#### 2.3.4 Teacher facilitation

We will send teachers a daily, 8-question survey asking about their facilitation of the program (see S1 File). We will also ask teachers to take daily photos of the motor skill activities on the provided tablet. This survey plus pictures is a proxy for intervention fidelity. Questions were designed to include 11 of the 12 TiDIER structures[76, 77]. Specifically, the survey includes information on the name of the program, materials, procedures, provider, mode of delivery, where/location, when/how much, tailoring, modifications, planned activities, and actual activities [76, 77]. Primary outcomes of interest will be teacher-reported facilitation as assessed as a percent of sessions and session components fully and correctly executed.

#### 2.3.5. e:Health platform feasibility, usability, and engagement

Feasbility and usability of the private YouTube channel will be assessed using the System Usability Scale [78–80]. Teachers in e:MAP classrooms will be asked to complete this survey three times: before the start of the program, one week after the start of e:MAP, and at the end of e:MAP. Engagement with the platform will be collected as the number of video views on e:MAP sessions weekly starting at the beginning of the intervention.

### 2.4 Timeline

**Fig 1:**
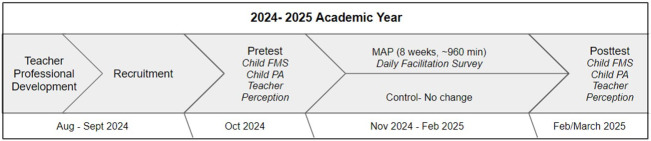
Study timeline and procedures.

This study will take place across the 2024–2025 school year. See Fig 1 for a full description of the study procedures and timeline.

### 2.5 Ethics approval

The Institutional Review Board at the University of Michigan has reviewed all study procedures. All study procedures involving collecting data from children have been approved (HUM00242675), and all study procedures involving teachers have been deemed exempt (HUM00252364).

### 2.6 Study procedures

This study will take place at a single early childhood center in the Midwestern United States during the 2024–2025 school year. The center is federally subsidized and provides high-quality early childhood education to children from low socioeconomic backgrounds. The center has agreed to partner on this research project and was consulted on all aspects of the study. The center will receive half of the equipment and be granted continued access to the program after the cessation of the project.

Before the start of the 2024–2025 school year, the research staff will attend the teacher professional development and provide a 1 to 2-hour overview of the project and the e:MAP program. In this session, we will review the purpose of e:MAP, the components of e:MAP, how to access and use the e:health platform, teacher facilitation expectations, and the recruitment plan. We will address any questions teachers or school staff have regarding the project. We will also be on-site once a month during the intervention period to address any concerns or assist with any technical difficulties. If teachers are not completing at least three e:MAP sessions per week, a member of the research team will contact them to offer additional support.

All teachers of classrooms with children who are eligible to enroll in the study will complete an initial survey on their perception of the programs (August 2024). Recruitment will start in August (see recruitment strategies #1 & #2) and September (recruitment strategy #3). Baseline testing of children’s outcomes (FMS and PA) will be completed in October 2024. Classrooms will then be randomly assigned to a MAP condition or a standard of care condition. Classrooms assigned to e:MAP will complete the intervention from November 2024 –February 2025. During this time, teachers whose classrooms are randomly assigned to the e:MAP program will facilitate the program by playing a daily e:MAP session video on a tablet provided to the classroom and setting up motor skills stations using the provided equipment and instructions. These teachers will also complete a daily facilitation survey. We will then complete post-test child health outcomes and surveys on teachers’ perceptions of MAP in February–March 2025.

### 2.7. Safety considerations and adverse events

This study does not pose any additional safety risks for children above what is already present in outdoor free play. However, should an issue arise, the school administration will report the event to the researchers using the Adverse Event Report Form (see S2 File). Researchers will report adverse events to the hosting IRB and follow IRB protocols. Adverse event forms include nine questions on (#1) the date of the event, (#2) a description of the event, (#3) the severity of the event [*mild; moderate; severe; life-threatening*], (#4) if the event was related to the study [*not related; unlikely related; possibly related; probably related; definitely related*], (#5–6) description of the action taken [*none; dose modification; medical intervention; hospitalization; intervention discontinued; other*], (#7) the outcome of the event [*resolved; recovered with minor sequelae; recovered with major sequelae; ongoing/continuing treatment; condition worsening; death; unknown*], (#8) if the adverse event was expected [*yes; no*], and (#9) if this was a serious event [*yes; no*]. If the event is categorized as a serious event, a second form will be completed (form link: https://www.nia.nih.gov/sites/default/files/adverse_events_form.pdf).

### 2.8 Planned analyses

Whole-study and group-specific descriptive statistics, including frequencies and percentages of categorical variables, along with mean and standard deviation of continuous variables, will be computed to summarize the sample characteristics. Between-group comparisons will be conducted at baseline to evaluate the success of randomization using two-sample t-tests. The post-intervention trajectory of FMS and PA will be assessed for any group differences, and we will use multilevel linear mixed effect models or generalized linear mixed effect models to determine covariate-adjusted differences between the MAP group and control group after the intervention, controlling for the established influence of sex, age, and anthropometrics on child outcomes [10, 70, 71]. Besides the main effect of each variable, the interaction term between intervention and sex will also be evaluated to investigate whether the intervention had a differential effect for boys and girls.

In regard to teacher outcomes, we will use a dependent sample t-test to explore if there are changes in teachers’ perceptions of the program at posttest. Teacher’s ability to facilitate the program will be reported as a percentage of sessions where teachers correctly executed MAP.

## 3. Conclusion and future directions

To the best of our knowledge, this work represents the first e:health intervention using Achievement Goal Theory to be implemented within an early childhood education center. The results of this study will provide novel insight into the e:health modality for FMS and PA interventions in this context. We acknowledge this work is not without limitations, such as recruiting from only one center and only including two time points of data collection. These decisions were made based on project feasibility, the requirements of the funders, and recommendations from community partners. Lastly, we recognize we are creating a pseudo-mastery not a full mastery climate. This decision aligned with previous work [45] and was made intentionally to best address known barriers around school interventions [31]; however, it may limit the comparability of results to full-mastery climates.

While not a limitation, it is worth noting we selected to ground our work in Achievement Goal Theory and designed the two aspects of the program (i.e., videos and activities) based on the tenets of this theory. This was one approach, and we recognized that other theories could also be very powerful to utilize in e:health programs. For example, future programs could consider developing their work based on Self-Determination Theory and design their e:health or m:health program based on identifiable teacher motivational behaviors recommended for these types of interventions [81]. We recommend future work build upon this initial investigation and continue to examine the effects of this or similar e:health interventions in larger samples, using a longer study timeline with retention tests, or using different/diverse theoretical approaches in their design.

Even with these limitations, completing this work will yield new knowledge. Specifically, these results will provide insight into how e:health movement interventions could be used to promote health in children at risk for future health disparities. These results will also provide insight into the potential for e:health interventions as an effective and potentially sustainable/distributable intervention modality. This work will provide much-needed insight into the feasibility/usability, teachers’ perceptions, and teachers’ ability to facilitate e:health programs.

## Supporting information

S1 File Daily MAP e:Health facilitation survey. (PDF)

S2 File Adverse event form (e:MAP). (PDF)

## References

[1] Global status report on physical activity 2022. World Health Organization; 2022. https://www.who.int/teams/health-promotion/physical-activity/global-status-report-on-physical-activity-2022

[2] Santos AC, Willumsen J, Meheus F, Ilbawi A, Bull FC. The cost of inaction on physical inactivity to public health-care systems: a population-attributable fraction analysis. Lancet Glob Health. 2023;11: e32–e39. doi: 10.1016/S2214-109X(22)00464-8 36480931 PMC9748301

[3] Beets MW, Bornstein D, Dowda M, Pate RR. Compliance With National Guidelines for Physical Activity in U.S. Preschoolers: Measurement and Interpretation. Pediatrics. 2011;127: 658–664. doi: 10.1542/peds.2010-2021 21422082 PMC3387888

[4] Friel CP, Duran AT, Shechter A, Diaz KM. U.S. Children Meeting Physical Activity, Screen Time, and Sleep Guidelines. Am J Prev Med. 2020;59: 513–521. doi: 10.1016/j.amepre.2020.05.007 32863080 PMC7574791

[5] Tapia-Serrano MA, Sevil-Serrano J, Sánchez-Miguel PA, López-Gil JF, Tremblay MS, García-Hermoso A. Prevalence of meeting 24-Hour Movement Guidelines from pre-school to adolescence: A systematic review and meta-analysis including 387,437 participants and 23 countries. J Sport Health Sci. 2022;11: 427–437. doi: 10.1016/j.jshs.2022.01.005 35066216 PMC9338333

[6] American Health Rankings. United Health Foundation 2022; 2022.

[7] Clark J, Metcalfe J S. The mountain of motor development: a metaphor. Mot Dev Res Rev. 2002;2: 183–202.

[8] Seefeldt V. Developmental motor patterns: implications for elementary school physical fitness. In: Nadeau CH, Halliwell WR, Newell KC, Roberts GC, editors. Psychology of motor behavior and sport. Champaign, IL: Human Kinetic; 1980. pp. 314–323.

[9] Goodway J, Ozmun JC, Gallahue DL. Understanding motor development: infants, children, adolescents, adults. Eighth edition. Burlington, MA: Jones & Bartlett Learning; 2021.

[10] Ulrich, D A. TGMD-3: Test of Gross Motor Development-Third Edition. Pro-Ed; 2019.

[11] Stodden DF, Goodway JD, Langendorfer SJ, Roberton MA, Rudisill ME, Garcia C, et al. A Developmental Perspective on the Role of Motor Skill Competence in Physical Activity: An Emergent Relationship. Quest. 2008;60: 290–306. doi: 10.1080/00336297.2008.10483582

[12] Logan SW, Kipling Webster E, Getchell N, Pfeiffer KA, Robinson LE. Relationship Between Fundamental Motor Skill Competence and Physical Activity During Childhood and Adolescence: A Systematic Review. Kinesiol Rev. 2015;4: 416–426. doi: 10.1123/kr.2013-0012

[13] Lima RA, Pfeiffer K, Larsen LR, Bugge A, Moller NC, Anderson LB, et al. Physical Activity and Motor Competence Present a Positive Reciprocal Longitudinal Relationship Across Childhood and Early Adolescence. J Phys Act Health. 2017;14: 440–447. doi: 10.1123/jpah.2016-0473 28169569

[14] Robinson LE, Stodden DF, Barnett LM, Lopes VP, Logan SW, Rodrigues LP, et al. Motor Competence and its Effect on Positive Developmental Trajectories of Health. Sports Med. 2015;45: 1273–1284. doi: 10.1007/s40279-015-0351-6 26201678

[15] Barnett LM, Webster EK, Hulteen RM, De Meester A, Valentini NC, Lenoir M, et al. Through the Looking Glass: A Systematic Review of Longitudinal Evidence, Providing New Insight for Motor Competence and Health. Sports Med. 2022;52: 875–920. doi: 10.1007/s40279-021-01516-8 34463945 PMC8938405

[16] Cliff DP, Okely AD, Smith LM, McKeen K. Relationships between Fundamental Movement Skills and Objectively Measured Physical Activity in Preschool Children. Pediatr Exerc Sci. 2009;21: 436–449. doi: 10.1123/pes.21.4.436 20128363

[17] Stodden D, Goodway JD. The Dynamic Association Between Motor Skill Development and Physical Activity. J Phys Educ Recreat Dance. 2007;78: 33–49. doi: 10.1080/07303084.2007.10598077

[18] Logan SW, Robinson LE, Wilson AE, Lucas WA. Getting the fundamentals of movement: a meta-analysis of the effectiveness of motor skill interventions in children. Child Care Health Dev. 2012;38: 305–315. doi: 10.1111/j.1365-2214.2011.01307.x 21880055

[19] Brian A, Pennell A, Taunton S, Starrett A, Howard-Shaughnessy C, Goodway JD, et al. Motor Competence Levels and Developmental Delay in Early Childhood: A Multicenter Cross-Sectional Study Conducted in the USA. Sports Med. 2019;49: 1609–1618. doi: 10.1007/s40279-019-01150-5 31301035

[20] Chen Z, Zhu W, Ulrich DA, Qin M. Have the Fundamental Movement Skills of U.S. Children Changed? Res Q Exerc Sport. 2024;95: 431–440. doi: 10.1080/02701367.2023.2250828 37801711

[21] Bolger LE, Bolger LA, O’Neill C, Coughlan E, O’Brien W, Lacey S, et al. Global levels of fundamental motor skills in children: A systematic review. J Sports Sci. 2021;39: 717–753. doi: 10.1080/02640414.2020.1841405 33377417

[22] Wick K, Leeger-Aschmann CS, Monn ND, Radtke T, Ott LV, Rebholz CE, et al. Interventions to Promote Fundamental Movement Skills in Childcare and Kindergarten: A Systematic Review and Meta-Analysis. Sports Med. 2017;47: 2045–2068. doi: 10.1007/s40279-017-0723-1 28386652 PMC5603621

[23] Palmer KK, Chinn KM, Robinson LE. Using Achievement Goal Theory in Motor Skill Instruction: A Systematic Review. Sports Med. 2017;47: 2569–2583. doi: 10.1007/s40279-017-0767-2 28779359

[24] Van Capelle A, Broderick CR, Van Doorn N, E. Ward R, Parmenter BJ. Interventions to improve fundamental motor skills in pre-school aged children: A systematic review and meta-analysis. J Sci Med Sport. 2017;20: 658–666. doi: 10.1016/j.jsams.2016.11.008 28169146

[25] Van Der Walt J, Plastow NA, Unger M. Motor skill intervention for pre-school children: A scoping review. Afr J Disabil. 2020;9. doi: 10.4102/ajod.v9i0.747 33354535 PMC7736652

[26] Khodaverdi Z, O’Brien W, Duncan M, Clark CCT. Motor competence interventions in children and adolescents–theoretical and atheoretical approaches: A systematic review. J Sports Sci. 2022;40: 2233–2266. doi: 10.1080/02640414.2022.2148897 36469747

[27] Lander N, Eather N, Morgan PJ, Salmon J, Barnett LM. Characteristics of Teacher Training in School-Based Physical Education Interventions to Improve Fundamental Movement Skills and/or Physical Activity: A Systematic Review. Sports Med. 2017;47: 135–161. doi: 10.1007/s40279-016-0561-6 27294354

[28] Robinson LE, Wang L, Colabianchi N, Stodden DF, Ulrich D. Protocol for a two-cohort randomized cluster clinical trial of a motor skills intervention: The Promoting Activity and Trajectories of Health (PATH) Study. BMJ Open. 2020;10: e037497. doi: 10.1136/bmjopen-2020-037497 32532781 PMC7295413

[29] Robinson LE, Palmer KK, Santiago-Rodríguez ME, Myers ND, Wang L, Pfeiffer KA. Protocol for a multicenter-cluster randomized clinical trial of a motor skills intervention to promote physical activity and health in children: the CHAMP afterschool program study. BMC Public Health. 2022;22: 1544. doi: 10.1186/s12889-022-13849-8 35964114 PMC9375271

[30] Brian A, Pennell A, Sacko R, Schenkelburg M. Preschool Teachers’ Preparedness for Knowing, Enabling, and Meeting the Active Start Guidelines for Physical Activity. J Mot Learn Dev. 2018;6: 333–344. doi: 10.1123/jmld.2017-0033

[31] Jerebine A, Heering T, Barnett LM. Educator-Perceived Barriers and Facilitators to Structured-Physical Activity in Early Childhood Centres: A Systematic Review. Res Q Exerc Sport. 2024;95: 243–262. doi: 10.1080/02701367.2023.2193243 37327492

[32] LaPlante C, Peng W. A Systematic Review of e-Health Interventions for Physical Activity: An Analysis of Study Design, Intervention Characteristics, and Outcomes. Telemed E-Health. 2011;17: 509–523. doi: 10.1089/tmj.2011.0013 21718092

[33] Swindle T, Poosala AB, Zeng N, Børsheim E, Andres A, Bellows LL. Digital Intervention Strategies for Increasing Physical Activity Among Preschoolers: Systematic Review. J Med Internet Res. 2022;24: e28230. doi: 10.2196/28230 35014962 PMC8790686

[34] Nyström CD, Sandin S, Henriksson P, Henriksson H, Trolle-Lagerros Y, Larsson C, et al. Mobile-based intervention intended to stop obesity in preschool-aged children: the MINISTOP randomized controlled trial,. Am J Clin Nutr. 2017;105: 1327–1335. doi: 10.3945/ajcn.116.150995 28446496

[35] Webster EK, Kracht CL, Newton RL, Beyl RA, Staiano AE. Intervention to Improve Preschool Children’s Fundamental Motor Skills: Protocol for a Parent-Focused, Mobile App-Based Comparative Effectiveness Trial. JMIR Res Protoc. 2020;9: e19943. doi: 10.2196/19943 33079066 PMC7609200

[36] Staiano AE, Newton RL, Beyl RA, Kracht CL, Hendrick CA, Viverito M, et al. mHealth Intervention for Motor Skills: A Randomized Controlled Trial. Pediatrics. 2022;149: e2021053362. doi: 10.1542/peds.2021-053362 35415743 PMC9648112

[37] Champion KE, Parmenter B, McGowan C, Spring B, Wafford QE, Gardner LA, et al. Effectiveness of school-based eHealth interventions to prevent multiple lifestyle risk behaviours among adolescents: a systematic review and meta-analysis. Lancet Digit Health. 2019;1: e206–e221. doi: 10.1016/S2589-7500(19)30088-3 33323269

[38] Elliott ES, Dweck CS. Goals: An approach to motivation and achievement. J Pers Soc Psychol. 1988;54: 5–12. doi: 10.1037//0022-3514.54.1.5 3346808

[39] Nicholls JG, Patashnick M, Nolen SB. Adolescents’ theories of education. J Educ Psychol. 1985;77: 683–692. doi: 10.1037/0022-0663.77.6.683

[40] Meece JL, Blumenfeld PC, Hoyle RH. Students’ goal orientations and cognitive engagement in classroom activities. J Educ Psychol. 1988;80: 514–523. doi: 10.1037/0022-0663.80.4.514

[41] Ames C, Archer J. Achievement goals in the classroom: Students’ learning strategies and motivation processes. J Educ Psychol. 1988;80: 260–267. doi: 10.1037/0022-0663.80.3.260

[42] Robinson LE, Rudisill ME, Goodway JD. Instructional Climates in Preschool Children Who Are At-Risk. Part II: Perceived Physical Competence. Res Q Exerc Sport. 2009;80: 543–551. doi: 10.1080/02701367.2009.10599592 19791640

[43] Robinson LE, Palmer KK, Bub KL. Effect of the Children’s Health Activity Motor Program on Motor Skills and Self-Regulation in Head Start Preschoolers: An Efficacy Trial. Front Public Health. 2016;4. doi: 10.3389/fpubh.2016.00173 27660751 PMC5014876

[44] Miller AL, Palmer KK, Wang L, Wang C, Riley HO, McClelland MM, et al. Mastery-oriented motor competence intervention improves behavioral but not cognitive self-regulation in head start preschoolers: Randomized controlled trial results. Scand J Med Sci Sports. 2023;33: 725–736. doi: 10.1111/sms.14294 36577657 PMC10441036

[45] Palmer KK, Miller AL, Meehan SK, Robinson LE. The Motor skills At Playtime intervention improves children’s locomotor skills: A feasibility study. Child Care Health Dev. 2020;46: 599–606. doi: 10.1111/cch.12793 32557838 PMC8218890

[46] Morley D, Till K, Ogilvie P, Turner G. Influences of gender and socioeconomic status on the motor proficiency of children in the UK. Hum Mov Sci. 2015;44: 150–156. doi: 10.1016/j.humov.2015.08.022 26342797

[47] Gosselin V, Leone M, Laberge S. Socioeconomic and gender-based disparities in the motor competence of school-age children. J Sports Sci. 2021;39: 341–350. doi: 10.1080/02640414.2020.1822585 32967566

[48] Baharvand P, Nejad EB, Karami K, Amraei M. A Review Study of the Role of Socioeconomic Status and its Components in Children’s Health. Glob J Med Pharm Biomed Update. 2021;16: 9. doi: 10.25259/GJMPBU_10_2021

[49] Poulton R, Caspi A, Milne BJ, Thomson WM, Taylor A, Sears MR, et al. Association between children’s experience of socioeconomic disadvantage and adult health: a life-course study. The Lancet. 2002;360: 1640–1645. doi: 10.1016/S0140-6736(02)11602-3 12457787 PMC3752775

[50] National Physical Activity Plan Alliance. The 2018 United States report card on physical activity for children and youth. Washington (DC): National Physical Activity Plan Alliance; 2019.

[51] Williams AS, Ge B, Petroski G, Kruse RL, McElroy JA, Koopman RJ. Socioeconomic Status and Other Factors Associated with Childhood Obesity. J Am Board Fam Med. 2018;31: 514–521. doi: 10.3122/jabfm.2018.04.170261 29986976 PMC9118515

[52] Barriuso L, Miqueleiz E, Albaladejo R, Villanueva R, Santos JM, Regidor E. Socioeconomic position and childhood-adolescent weight status in rich countries: a systematic review, 1990–2013. BMC Pediatr. 2015;15: 129. doi: 10.1186/s12887-015-0443-3 26391227 PMC4578240

[53] Milaniak I, Jaffee SR. Childhood socioeconomic status and inflammation: A systematic review and meta-analysis. Brain Behav Immun. 2019;78: 161–176. doi: 10.1016/j.bbi.2019.01.018 30738842

[54] Reardon SF. The widening academic achievement gap between the rich and the poor: New evidence and possible explanations. In: Duncan GJ, Murnane RJ, editors. Whither Opportunity?: Rising Inequality, Schools, and Children’s Life Chances. Stanford University, CA: Russell Sage Foundation; 2011. pp. 91–116.

[55] Bradley RH, Corwyn RF. Socioeconomic Status and Child Development. Annu Rev Psychol. 2002;53: 371–399. doi: 10.1146/annurev.psych.53.100901.135233 11752490

[56] Garcia, Emma, Weiss, Elaine. Education Inequalities at the School Starting Gate: Gaps, Trends, and Strategies to Address Them. Washington (DC): Economic Policy Institute; 2017.

[57] Swift DL, Johannsen NM, Lavie CJ, Earnest CP, Church TS. The Role of Exercise and Physical Activity in Weight Loss and Maintenance. Prog Cardiovasc Dis. 2014;56: 441–447. doi: 10.1016/j.pcad.2013.09.012 24438736 PMC3925973

[58] Lee I-M. Physical Activity and Weight Gain Prevention. JAMA. 2010;303: 1173. doi: 10.1001/jama.2010.312 20332403 PMC2846540

[59] Ertek S, Cicero A. Impact of physical activity on inflammation: effects on cardiovascular disease risk and other inflammatory conditions. Arch Med Sci. 2012;5: 794–804. doi: 10.5114/aoms.2012.31614 23185187 PMC3506236

[60] Andersen LB, Riddoch C, Kriemler S, Hills A. Physical activity and cardiovascular risk factors in children. Br J Sports Med. 2011;45: 871–876. doi: 10.1136/bjsports-2011-090333 21791456

[61] Haapala EA, Väistö J, Ihalainen JK, González CT, Leppänen MH, Veijalainen A, et al. Associations of physical activity, sedentary time, and diet quality with biomarkers of inflammation in children. Eur J Sport Sci. 2022;22: 906–915. doi: 10.1080/17461391.2021.1892830 33599556

[62] Eisenmann JC. Physical activity and cardiovascular disease risk factors in children and adolescents: an overview. Can J Cardiol. 2004;20: 295–301. 15054507

[63] Lean AS, Leon AS. Physical activity and cardiovascular health: a national consensus. Champaign, Ill.: Human Kinetics; 1997.

[64] Sample Size Calculator. https://clincalc.com/stats/samplesize.aspx

[65] Ames C. Classrooms: Goals, structures, and student motivation. J Educ Psychol. 1992;84: 261–271. doi: 10.1037/0022-0663.84.3.261

[66] Ames C. Achievement Goals, Motivational Climate, and Motivational Processes. In: Roberts G, editor. Motivation in Sport and Exercise. Champaign, IL: Human Kinetics; 1995. pp. 161–176.

[67] Epstein J L. Effective schools or effective students: Dealing with diversity. In: Haskins R, MacRae D, editors. Policies for America’s public schools: Teachers, equity, and indicators. New York (NY): Ablex Publishing; 1988. pp. 89–126.

[68] Morrison LG, Yardley L, Powell J, Michie S. What Design Features Are Used in Effective e-Health Interventions? A Review Using Techniques from Critical Interpretive Synthesis. Telemed E-Health. 2012;18: 137–144. doi: 10.1089/tmj.2011.0062 22381060

[69] Robinson LE, Palmer KK, Irwin JM, Webster EK, Dennis AL, Brock SJ, et al. The Use of Multimedia Demonstration on the Test of Gross Motor Development–Second Edition: Performance and Participant Preference. J Mot Learn Dev. 2015;3: 110–122. doi: 10.1123/jmld.2014-0064

[70] Palmer KK, Stodden DF, Ulrich DA, Robinson LE. Using Process- and Product-oriented Measures to Evaluate Changes in Motor Skills across an Intervention. Meas Phys Educ Exerc Sci. 2021;25: 273–282. doi: 10.1080/1091367X.2021.1876069 34354338 PMC8336534

[71] Palmer KK, Pennell A, Terlizzi B, Nunu MA, Stodden DF, Robinson LE. Performance Metrics From Product-Oriented Measures of Fundamental Motor Skills—A Comparison and Developmental Perspective. J Mot Learn Dev. 2023;11: 401–423. doi: 10.1123/jmld.2022-0074 38500698 PMC10947211

[72] Johansson E, Larisch L-M, Marcus C, Hagströmer M. Calibration and Validation of a Wrist- and Hip-Worn Actigraph Accelerometer in 4-Year-Old Children. Hind K, editor. PLOS ONE. 2016;11: e0162436. doi: 10.1371/journal.pone.0162436 27617962 PMC5019366

[73] Miller GD, Jakicic JM, Rejeski WJ, Whit-Glover MC, Lang W, Walkup MP, et al. Effect of varying accelerometry criteria on physical activity: The look ahead study. Obesity. 2013;21: 32–44. doi: 10.1002/oby.20234 23505166 PMC3430806

[74] Cain KL, Sallis JF, Conway TL, Van Dyck D, Calhoon L. Using Accelerometers in Youth Physical Activity Studies: A Review of Methods. J Phys Act Health. 2013;10: 437–450. doi: 10.1123/jpah.10.3.437 23620392 PMC6331211

[75] Webster-Stratton C, Jamila Reid M, Stoolmiller M. Preventing conduct problems and improving school readiness: evaluation of the Incredible Years Teacher and Child Training Programs in high-risk schools. J Child Psychol Psychiatry. 2008;49: 471–488. doi: 10.1111/j.1469-7610.2007.01861.x 18221346 PMC2735210

[76] Hoffmann TC, Glasziou PP, Boutron I, Milne R, Perera R, Moher D, et al. Better reporting of interventions: template for intervention description and replication (TIDieR) checklist and guide. BMJ. 2014;348: g1687–g1687. doi: 10.1136/bmj.g1687 24609605

[77] Cotterill S, Knowles S, Martindale A-M, Elvey R, Howard S, Coupe N, et al. Getting messier with TIDieR: embracing context and complexity in intervention reporting. BMC Med Res Methodol. 2018;18: 12. doi: 10.1186/s12874-017-0461-y 29347910 PMC5774137

[78] Brooke J. SUS: A “quick and dirty” usability scale. In: Jordan P W, Thomas B, Weerdmeester B A, McClelland I L, editors. Usability evaluation in industry. London: Taylor & Francis; 1996. pp. 189–194.

[79] Bangor A, Kortum PT, Miller JT. An Empirical Evaluation of the System Usability Scale. Int J Hum-Comput Interact. 2008;24: 574–594. doi: 10.1080/10447310802205776

[80] Lewis JR. The System Usability Scale: Past, Present, and Future. Int J Human–Computer Interact. 2018;34: 577–590. doi: 10.1080/10447318.2018.1455307

[81] Ahmadi A, Noetel M, Parker P, Ryan RM, Ntoumanis N, Reeve J, et al. A classification system for teachers’ motivational behaviors recommended in self-determination theory interventions. J Educ Psychol. 2023;115: 1158–1176. doi: 10.1037/edu0000783

