# Supplementary material for: Registered report protocol for an e: Health motor skills and physical activity intervention in early childhood education centers- e: Motor skills At Playtime (MAP)
Source: PLoS One. 2024 Aug 29;19(8):e0308047. doi: 10.1371/journal.pone.0308047 (PMC11361570; doi:10.1371/journal.pone.0308047)
Supplement: S1 File — (PDF) [file pone.0308047.s001.pdf]

Daily Facilitation of e:MAP Survey

Instructions: *Please complete the following survey about MAP today. NOTE: You will be asked if you took photos of the stations. Estimated Time: 5 minutes*

Did you use MAP today? ▢ Yes ▢ No

[answer determines branch logic below]

[if NO, then…

Did someone else provide MAP today? ▢ Yes ▢ No

If so, who? Who provided MAP:____________________

[if YES, then….

What session/video number did you use? Session/Video #__________

Was MAP delivered outside on the playground or in another location?

▢ Outside/Playground
▢ Gym/Indoor play space
▢ Other:__________

Did you set up the corresponding skill stations? ▢ Yes ▢ No

Did you take a photo of each skill station on the tablet provided? ▢ Yes ▢ No

[if NO, then…

What set up number from the box did you implement?

Skill name:________________________
Set Up Number: ____________________

Approximate Start Time/End Time

Start Time:__________
End Time:__________

Did you notice children using the MAP

station/equipment for the skills included in the MAP video?	▢ Yes	▢ No
Did you notice children using the MAP station/equipment for activities OTHER than the skills included in the MAP video?	▢ Yes	▢ No

[if YES, then …

Please briefly describe? Other station/equipment activities:___________
