# Supplementary material for: Registered report protocol for an e: Health motor skills and physical activity intervention in early childhood education centers- e: Motor skills At Playtime (MAP)
Source: PLoS One. 2024 Aug 29;19(8):e0308047. doi: 10.1371/journal.pone.0308047 (PMC11361570; doi:10.1371/journal.pone.0308047)
Supplement: S2 File — (PDF) [file pone.0308047.s002.pdf]

Adverse Event Form (e:MAP)

Date of the event: Click or tap to enter a date.

Description of the event:

Severity of the event: Choose an item.

Was this event related to the study? Choose an item.

Description of action taken:

Please select which best describes the action taken Choose an item.?

Outcome of the Adverse Event: Choose an item.

Was this adverse event expected? Choose an item.

Was this a Serious Adverse Event? Choose an item.
